# All-Nitrogen Energetic Material Cubic Gauche Polynitrogen: Plasma Synthesis and Thermal Performance

**DOI:** 10.3390/molecules29020504

**Published:** 2024-01-19

**Authors:** Chenxi Qu, Jiale Li, Kewei Ding, Songsong Guo, Yating Jia

**Affiliations:** 1 Xi’an Modern Chemistry Research Institute, Xi’an 710065, China; qcx204@163.com (C.Q.);; 2 School of Chemistry and Chemical Engineering, Beijing Institute of Technology, Beijing 100081, China

**Keywords:** all-nitrogen material, polynitrogen, PECVD, thermal decomposition performance, decomposition kinetics

## Abstract

Numerous theoretical calculations have demonstrated that polynitrogen with an extending polymeric network is an ultrahigh-energy all-nitrogen material. Typical samples, such as cubic gauche polynitrogen (cg-N), have been synthesized, but the thermal performance of polynitrogen has not been unambiguously determined. Herein, macroscopic samples of polynitrogen were synthesized utilizing a coated substrate, and their thermal decomposition behavior was investigated. Polynitrogen with carbon nanotubes was produced using a plasma-enhanced chemical vapor deposition method and characterized using infrared, Raman, X-ray diffraction X-ray photoelectron spectroscopy and transmission electron microscope. The results showed that the structure of the deposited polynitrogen was consistent with that of cg-N and the amount of deposition product obtained with coated substrates increased significantly. Differential scanning calorimetry (DSC) at various heating rates and TG-DSC-FTIR-MS analyses were performed. The thermal decomposition temperature of cg-N was determined to be 429 °C. The apparent activation energy (E_a_) of cg-N calculated by the Kissinger and Ozawa equations was 84.7 kJ/mol and 91.9 kJ/mol, respectively, with a pre-exponential constant (lnA_k_) of 12.8 min^−1^. In this study, cg-N was demonstrated to be an all-nitrogen material with good thermal stability and application potential to high-energy-density materials.

## 1. Introduction

All-nitrogen materials have emerged as an important avenue for the development of high-energy-density materials (HEDMs). There have been significant experimental and theoretical advances in all-nitrogen materials, which are considered to be a strategic field for energetic materials [1,2,3]. All-nitrogen materials release enormous quantities of energy during decomposition into nitrogen because of the significant energy differences among the N≡N triple bond (954 kJ/mol), N=N double bond (418 kJ/mol) and N–N single bond (160 kJ/mol) [4]. Theoretical calculations have demonstrated the favorable performance of all-nitrogen materials, such as cg-N polynitrogen with an extending polymeric network, Td-N_4_ nitrogen clusters and *cyclo*-N_5_^−^ pentazolate anions. First-principles simulations on cg-N polynitrogen have indicated a density of 3.9 g/cm^3^ and an energy 10.6 times that of octahydro-1,3,5,7-tetranitro-1,3,5,7-tetrazocine (HMX) [5]. The theoretical heat of formation of a Td-N_4_ nitrogen cluster was determined to be 761 kJ/mol [6]. The detonation velocity and pressure of LiN_5_ were 11,362 m/s and 40.9 GPa, respectively [7]. All-nitrogen materials offer the advantages of a high density, high enthalpy of formation, ultrahigh energy, and clean, nonpolluting blast and are therefore considered a new generation of HEDMs for use in explosives and propellants.

Different preparation methods typically produce all-nitrogen materials with different structures. Significant initial progress has been made in the chemical synthesis of all-nitrogen compounds since the end of the 20th century. Christe et al. successfully synthesized a marginally stable all-nitrogen ion, N_5_^+^[AsF_6_]^−^ in 1999, which was followed by successive synthesis of more pentazeniums, such as N_5_^+^[SbF_6_]^−^, and N_5_^+^[Sb_2_F_11_] [8,9]. In 2017, Hu et al. and Lu et al. reported the synthesis and characterization of an atmospherically stable *cyclo*-N_5_^−^ salt, (N_5_)_6_(H_3_O)_3_(NH_4_)_4_Cl [10,11]. Researchers have since synthesized a series of metallic or nonmetallic salts of N_5_^−^ via ion exchange reactions [12,13]. Compounds containing all-nitrogen structural fragments, such as N8 (1,1′-azobis-1,2,3-triazole), N10 (1,1′-azobis(5-methyltetrazole)) and N11 (1,1′-(triaz-1-ene-1,3-diyl)bis(1H-tetrazol-5-amine)), have been synthesized successively [14,15], providing references for the preparation of all-nitrogen materials.

Polynitrogen materials with extended polymeric network structures are typically synthesized under extreme conditions of pressure and temperature. Under high pressure, nitrogen molecules are converted into solid polynitrogen with various crystalline structures, such as cg (cubic gauche), Cmcm (Cmcm chain), and ch (cis-transchain) [16,17]. The development of the diamond anvil cell (DAC) experimental technique and laser heating technology enabled cubic gauche nitrogen (cg-N) samples to be obtained for the first time under extreme experimental conditions of more than 110 GPa and 2000 K by Eremets et al. [18] in 2004. The major interplanar distances of cg-N at 115.4 GPa were found to be 2.4434 Å, 1.7279 Å, 1.4103 Å, 1.2206 Å and 1.0918 Å, corresponding to Miller-indices (hkl) of (110), (200), (211), (220) and (310), respectively. The Raman peak of cg-N at high pressure was reported at approximately 830 cm^−1^ that shifted with decreasing pressure at a rate of 1.53~1.57 cm^−1^/GPa [19,20]. This method was used in subsequent studies to synthesize various polynitrogen samples. Additional polynitrogen structures, such as hexagonal layered polynitrogen (HLP-N, 244 GPa and 3000 K) [21] and black phosphorus polynitrogen (BP-N, 140 GPa and 2300 K) [22], were discovered with the experimental pressure and temperature variation. In 2014, Yoo et al. [23] discovered a layered polymeric structure polynitrogen (LP-N) at 150 GPa and 2000 K that had two colossal Raman bands at 1000 cm^−1^ and 1300 cm^−1^. Subsequently, Li et al. [24] observed the post-layered-polymeric nitrogen (PLP-N) by further heating the LP-N to above 2300 K at 161 GPa.

Some all-nitrogen phases, such as cyclic N5 rings and pseudobenzene N6 hexagonal rings, have also been discovered in studies on phase transitions at high pressures [25,26]. To decrease the preparation pressure of polynitrogens, several researchers have used nitrogen-rich compounds as alternative raw materials to nitrogen. Azide compounds, including NaN_3_ [27], CsN_3_ [28], and KN_3_ [29], were initially tested as substrates for high-pressure polynitrogen preparation. However, only high-pressure phases of azides, such as Phase I and Phase II of NaN_3_ in the 0 GPa to 50 GPa range, have been found [27]. Conditions of more than 100 GPa and 2000 K are still required to transform azides into an amorphous polynitrogen structure.

Various novel methods for polynitrogen preparation under atmospheric conditions have been recently reported. A series of nitrogen clusters, such as TiN_12_^+^ [30], ZrN_12_^+^ [31], VN_x_^+^(x = 8, 9, 10) [32], and LiN_x_^+^ (x = 2, 4, 6, 8) [33], have been obtained by laser ablation of nitrogen-rich compounds. Cyclic voltammetry (CV) has been used to synthesize polynitrogen N_8_^−^ ions on pristine and boron-doped graphene, as well as on multiwalled carbon nanotubes (CNTs) [34,35]. The use of plasma-enhanced chemical vapor deposition (PECVD) to produce cg-N has opened an avenue for the synthesis of cg-N under ambient conditions [36,37]. However, in-depth studies on the thermal behavior of polynitrogens have not yet been conducted.

In conclusion, theoretical calculations prove that the cg-N polynitrogen is a high-energy-density all-nitrogen material. Recent advances have also been made in preparing cg-N polynitrogen at atmospheric pressure. However, the thermal stability and thermal decomposition performance of cg-N polynitrogen, which are crucial for practical applications in the field of energy materials, have not been determined. In this study, atmospherically stable cg-N polynitrogen with multiwall carbon nanotubes (cg-N/CNT) was synthesized by plasma-enhanced chemical vapor deposition. The deposition duration was optimized, and the product structures were characterized. The thermal properties of the polynitrogen samples were characterized, and the decomposition kinetics of the samples were calculated to investigate the application potential of polynitrogens as HEDMs.

## 2. Results and Discussion

### 2.1. X-ray Photoelectron Spectroscopy

**Figure 1:**
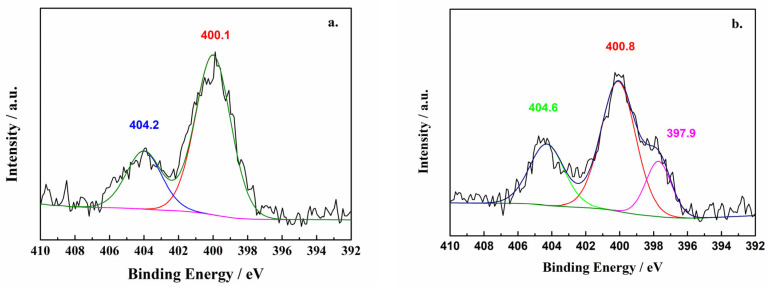
N 1s X-ray photoelectron spectrum of samples ((**a**) Before the deposition reaction, (**b**) after the deposition reaction).

The N 1s XPS spectrum of the NaN_3_/CNT substrate before and after the deposition reaction is shown in Figure 1. Figure 1a shows that the spectrum of the NaN_3_/CNT substrate exhibited two peaks at binding energies of 404.2 eV and 400.1 eV. Lorentz-Gaussian fitting yielded a peak ratio of approximately 1:2. These two peaks were thus determined to be generated by the azide ion of the raw material [36]. Figure 1b shows the N 1s XPS spectrum of the product of the deposition reaction. The two peaks with binding energies of 404.6 eV and 400.8 eV correspond to unreacted sodium azide. The slightly higher binding energies of the peaks compared to those in the spectrum obtained before deposition indicates that the sodium azide substrate may have undergone a solid-state phase change during deposition, but the chemical environment of the azide ion did not change significantly. Of particular interest is the peak with a binding energy of 397.9 eV, which does not lie either within the oxynitride binding energy interval or the interval for carbonitrides, such as pyridine or pyrrole [38]. Consequently, the 397.9 eV binding energy was determined to correspond to a single N–N bond and a similar binding energy to that determined by Benchafia et al. [36,39], confirming the presence of a nitrogenous product with a new valence and the same chemical environment for each nitrogen atom.

### 2.2. ATR-FTIR and Raman Spectroscopy

**Figure 2:**
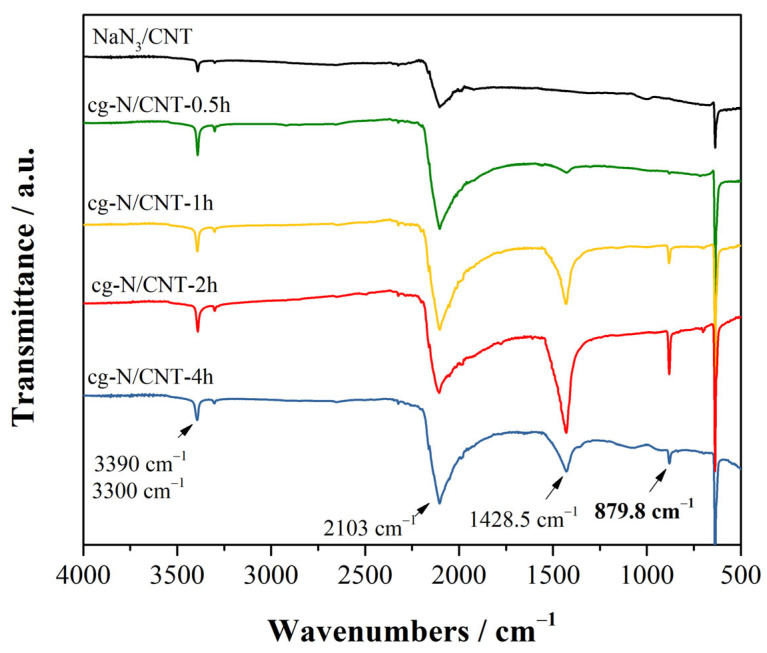
ATR-FTIR spectra of the samples before and after deposition at various reaction times.

Both IR and Raman are valuable for identifying changes in the azide groups during the deposition process because azide ions have active bending and asymmetric stretch modes. Figure 2 shows the IR spectra of the samples of the substrate and products of deposition with various reaction durations. The IR peaks at 2103 cm^−1^ and 638 cm^−1^ were attributed to the ν2 mode and anti-symmetric ν3 mode of the azide anion. The peaks at approximately 3300 cm^−1^ and 3390 cm^−1^ were assigned to the combination frequency vibration of the 2ν2+ν3 mode. The spectrum of the sample obtained after plasma deposition exhibited two prominent new peaks at approximately 1428 cm^−1^ and 879.8 cm^−1^. The infrared peak at 879.8 cm^−1^ was determined to be the absorption peak of cg-N at close to ambient pressure because of being identical to the T(TO) mode infrared absorption peak at near zero pressure produced in theoretical simulations carried out by Caracas et al. [40]. The prominent absorption peak near 1428 cm^−1^ was assigned to NaN_3_ Phase I at atmospheric pressure [27]. The infrared spectrum demonstrated that absorption of cg-N increased for two hours before decreasing. This result may have been caused by an increase in the atmospheric temperature during the deposition process that made the products disintegrate. Therefore, we selected a deposition duration of 2 h to ensure a sufficient quantity of product was obtained.

**Figure 3:**
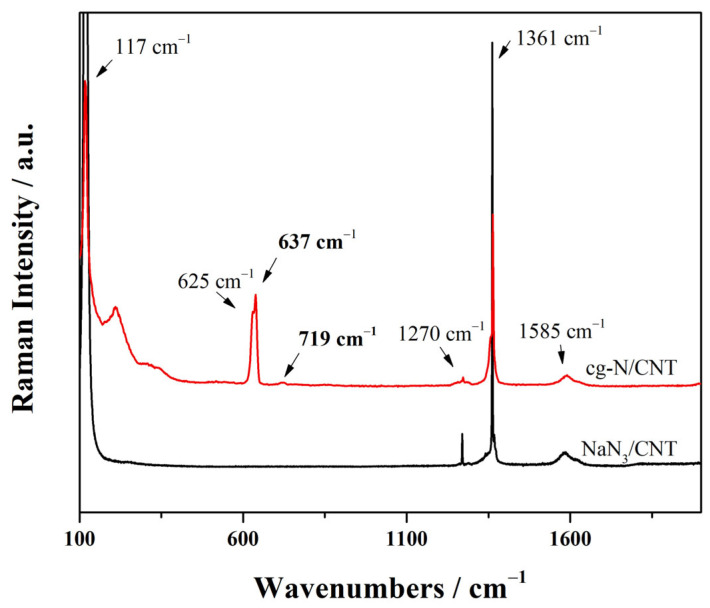
Raman spectra of the samples before and after deposition.

The Raman spectra of the plasma samples before and after the two-hour deposition reaction are shown in Figure 3. The Raman peaks of NaN_3_/CNT are shown by the black line. The sharp peak near 1340 cm^−1^ and the peaks near 1585 cm^−1^ were assigned to the D- and G-modes of the carbon nanotubes, respectively. The librational lattice mode, the IR active bending ν2 mode, and the symmetric stretching ν1 mode of the unreacted azide anion were characterized by three distinct, intense peaks at wavelengths of 117 cm^−1^, 1270 cm^−1^, and 1361 cm^−1^, respectively [25]. The red line indicates the spectrum obtained after the deposition reaction: the new broad peak at 637 cm^−1^ and the weaker peak at 719 cm^−1^ correspond to the A mode and tilted vibration (T-TO) mode Raman absorption estimated by Caracas [40]. The A-mode Raman peak of cg-N has also been experimentally observed at high pressure, where the peak is shifted considerably toward lower wavenumbers as the pressure decreases [18,19,20]. Therefore, the broad peak at 637 cm^−1^ and the sharp peak at 719 cm^−1^ in the spectrum of the deposition sample were determined to be the A-mode and T-TO mode Raman peaks under the zero-pressure condition of cg-N, respectively. The broad peaks at 210 cm^−1^ and 625 cm^−1^ were assigned to the nitrogen network in Phase I of NaN_3_ at high pressure and temperature [27]. By comparing the Raman peak fitting profiles of cg-N, the peak intensity ratio of cg-N to Phase I of NaN_3_ in the product obtained from the coated substrate was 0.75, while that of the product obtained from the powder substrate was 0.25. Preparation of the deposition substrate using the coating method increased the content of the polymerized nitrogen product nearly threefold.

The three NaN_3_ peaks are also considerably broader and weaker in intensity in the spectrum of the samples obtained after deposition than those obtained before deposition, indicating the dissociation of the azide anion and the formation of a nonmolecular nitrogen network during the reaction. The simultaneous increase in the I_d_/I_g_ ratio for the CNT peaks from 0.9 to 1.6 implies a significant increase in the defect density of CNTs. The stabilizing effect of the carbon tubes on the internal cg-N is reduced, indicating that the growth of cg-N during deposition results in an increase in carbon tube defects. This result could also be a contributing factor to the rapid depletion of cg-N over time.

### 2.3. X-ray Powder Diffraction

**Figure 4:**
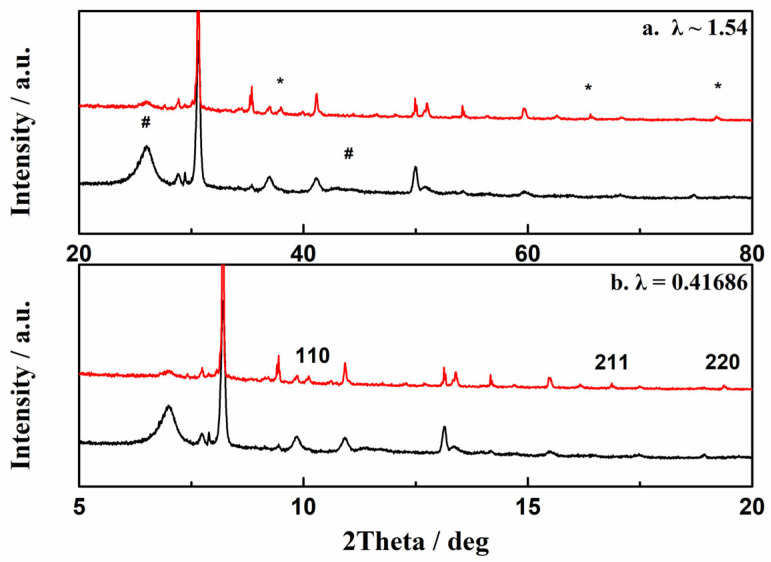
X-ray powder diffraction patterns of plasma deposition samples. (**a**) Diffraction patterns taken with λ ~ 1.54 Å, (**b**) diffraction pattern converted to λ = 0.41686 Å, (*) peaks of cg-N, (#) peaks of CNT.

Figure 4 presents the XRD patterns obtained for the samples before (black line) and after (red line) the plasma deposition reaction. In Figure 4a, the broad peaks (indicated by #) at approximately 26° and 45° are the diffraction peaks of the (002) and (110) crystal planes of the carbon nanotubes [41]. The series of strong peaks near 28°, 31°, and 36° correspond to unreacted sodium azide. Three prominent new reflection peaks (indicated by *) appear near 37°, 66°, and 77° in the spectrum of the sample obtained after plasma deposition. These peaks are considerably weaker than the diffraction peaks of NaN_3_ because of the lower crystallinity of the deposition products. Figure 4b shows the diffraction pattern of the sample converted to a wavelength λ = 0.41686 Å, which is the parameter in the diffraction cubic gauche polynitrogen reflection data measured by Eremets et al. [18] The bulk modulus of cg-N can reach 261 GPa at atmospheric pressure [42], which by far exceeds that of conventional ultrahard materials, such as silicon carbide. Therefore, the lattice spacings of cg-N do not change significantly at atmospheric pressure. The three diffraction peaks mentioned above were transformed into 2ndeta values of 9.8, 17.0, and 19.7. Comparison with the diffraction data of cg-N at 115 GPa shows that these new peaks are reflections by the (110), (211), and (220) crystal planes of cg-N. However, the relative intensities of these diffraction peaks are slightly different from those of cg-N, probably because of the crystallographic orientation induced by the carbon nanotubes.

### 2.4. Transmission Electron Microscopy

**Figure 5:**
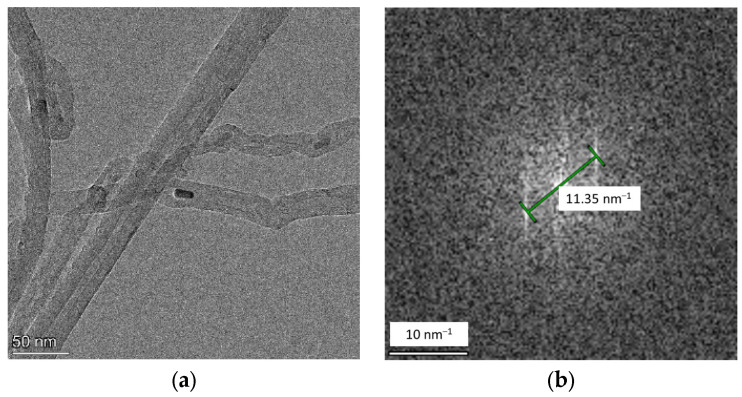
(**a**) High-resolution TEM images and (**b**) selected area electron diffraction (FFT) images of samples after deposition.

TEM images and selected area electron diffraction (FFT) images of the plasma deposited samples are shown in Figure 5. In the high-resolution TEM images, the deposited polynitrogen with a diameter of approximately 20–30 nm is distributed inside the multiwalled carbon nanotubes. Figure 5b shows the fast Fourier transform (FFT) image of the polynitrogen samples, from which the crystal plane spacing is measured as 1.75 Å. This crystal plane spacing is very close to the (200) plane spacing of 1.73 Å for cg-N obtained by Eremets et al. [18]. However, polynitrogen samples are considerably amorphous because plasma-deposited samples have low crystallinity, which is consistent with the XRD data.

### 2.5. Thermal Decomposition Performance of cg-N/CNT

**Figure 6:**
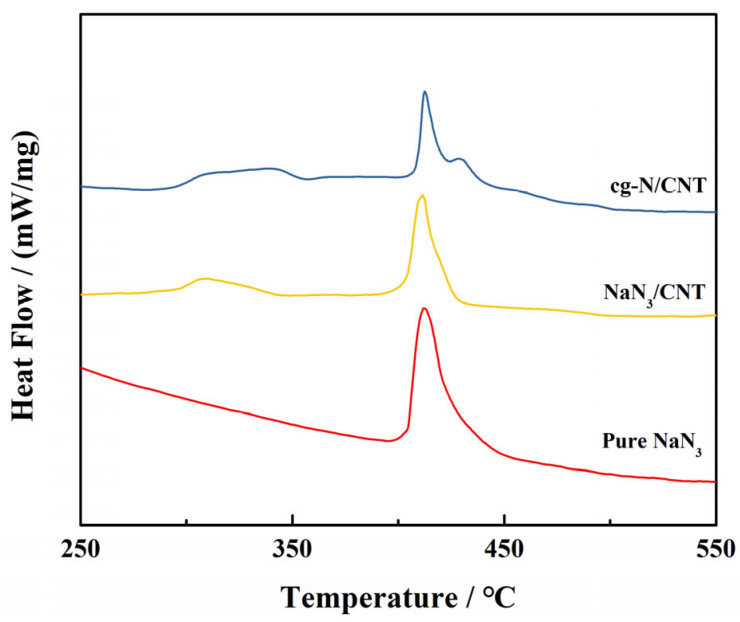
DSC curves of pure NaN_3_, NaN_3_/CNT and cg-N/CNT obtained at a heating rate of 10 °C/min.

To characterize the thermal stability of the samples, we measured the thermal decomposition temperature of the cg-N/CNT sample and its substrates using differential scanning calorimetry (DSC). Figure 6 presents the DSC curves obtained at a heating rate of 10 °C/min for the pure NaN_3_, NaN_3_/CNT and cg-N/CNT samples. The decomposition peak of pure sodium azide occurs at 412 °C, which is in reasonable agreement with that reported in the literature [43,44]. A broad exothermic peak between 275 °C and 350 °C appears for the NaN_3_/CNT sample obtained by compounding sodium azide with carbon nanotubes. The interaction of the carbon nanotubes with NaN_3_ in the interior decreases the thermal stability and therefore the thermal decomposition temperature of NaN_3_. There are two prominent exothermic peaks in the DSC curves of cg-N/CNT. The thermal decomposition peak of cg-N obtained by deposition occurs at approximately 429 °C. This result indicates that cg-N/CNT has superior thermal stability to the other two samples and a similar decomposition temperature to that determined using temperature programmed desorption (TPD) by Zhuang et al. [37]. Theoretical calculations suggest that carbon nanotubes stabilize polynitrogens, such as N_8_ oligomers [35], and may also contribute to the high thermal decomposition temperature of cg-N.

### 2.6. Non-Isothermal Decomposition Kinetics

To further analyze the behavior of the different components of the sample during thermal decomposition, the nonisothermal kinetic parameters of the exothermic decomposition reaction were investigated. The apparent activation energy (*E_a_*) was calculated using the Kissinger [45] and Ozawa [46] equations shown below:

(1) $$\ln\left(\frac{\beta }{T_{p}^{2}}\right)=\ln\left(\frac{A_{k}R}{E_{k}}\right)-\frac{E_{k}}{RT_{p}}$$

(2) $$\mathrm{lg}\beta +\frac{0.4567E_{o}}{RT_{p}}=C$$

where *β* is the heating rate for the sample, *R* is the gas constant (commonly taken as 8.3145 J/mol·K), and *T_p_* is the exothermic peak determined using the heating rate mentioned above. The apparent activation energy (*E_a_*) of the reaction can be calculated from the slope of the linear fit between *β* and 1/*T_p_* for the different equations. The preexponential constant (*A_k_*) can be obtained from the intercepts of the plots.

**Figure 7:**
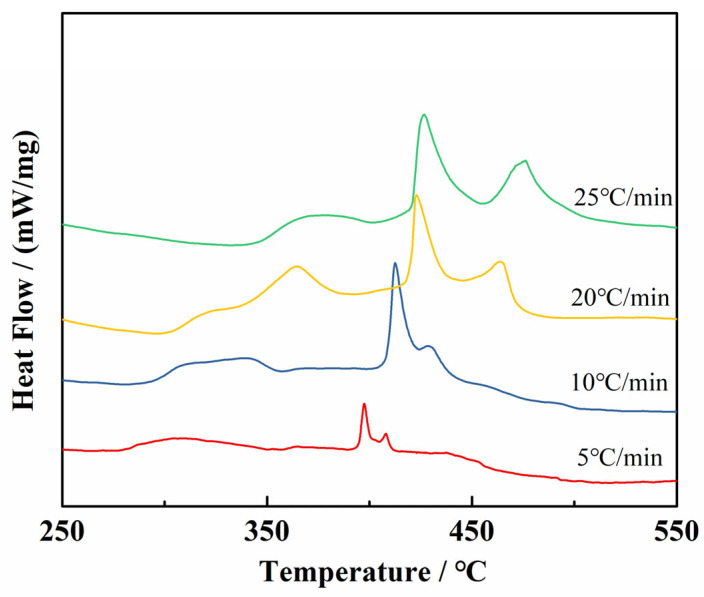
DSC curves of cg-N/CNT obtained at different heating rates.

The DSC measurements obtained at different heating rates are shown in Figure 7, and Table 1 shows the kinetic parameters of cg-N/CNT determined for heating rates of 5, 10, 20 and 25 °C/min. Substituting these parameters into the Kissinger and Ozawa equations yields essentially equivalent *E_a_* values. The apparent activation energy of cg-N/CNT obtained by the Kissinger method is 84.7 kJ/mol, similar to the 91.9 kJ/mol obtained by the Ozawa method. The pre-exponential factor of cg-N/CNT is 12.8 min^−1^. Furthermore, the apparent activation energy of cg-N/CNT are lower than that of NaN_3_, which implies that cg-N has a higher thermal stability and decomposition rate above the decomposition temperature than NaN_3_.

### 2.7. TG-DSC-FTIR-MS Analysis

**Figure 8:**
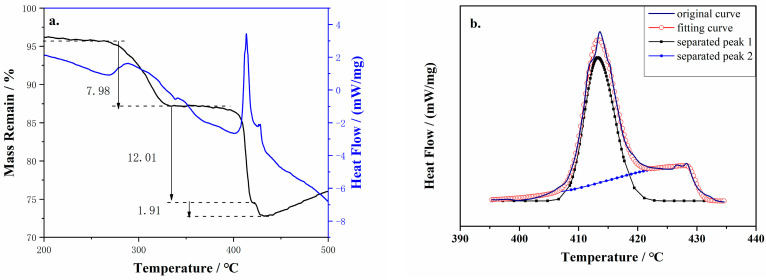
(**a**) TG−DSC curves and (**b**) fitted peaks for the cg-N/CNT samples obtained at a heating rate of 10 °C/min.

DSC-TG-FTIR-MS analysis was performed to characterize the heat released and the composition of the volatile gas released during the thermal decomposition of cg-N/CNT. Figure 8 shows the TG-DSC curve of cg-N/CNT obtained at a heating rate of 10 °C/min. Three significant thermal weight loss processes can be observed during the decomposition of the sample from room temperature to complete decomposition. The decomposition of NaN_3_ in the carbon nanotubes occurs between 275 and 350 °C, corresponding to the initial stage with an approximately 7.98 percent weight loss. The decomposition of the individual sodium azide crystals begins around 400 °C, which corresponds to the second stage with a weight loss of 12.01 percent. The disintegration of cg-N in the carbon nanotubes corresponds to the third stage of thermal weight loss, which starts at approximately 430 °C and results in a mass decrease of approximately 1.91 percent. Whereas XPS can only be used to characterize the surface of a sample, the entire sample is characterized by thermal analysis; thus, a significantly smaller mass percentage of cg-N is determined by thermal analysis than by XPS. The original exothermic peaks were divided into two peaks based on a Bigaussian function [47] to determine the ratios of the quantity of heat released by the various components, where the curves with the fitted peaks are presented in Figure 8b. Comparing the integration areas of the different curves results in an almost 3:2 ratio for the heat released by cg-N to that of NaN_3_ with the CNT substrate in an argon gas atmosphere. Considering that the mass ratio of cg-N to NaN_3_ is 6.3:1, the heat released per unit mass by cg-N is more than four times that of NaN_3_. Although the proportion may not be precise due to the small quantity of products tested, the heat release of cg-N polynitrogen is considerably higher than that of NaN_3_.

**Figure 9:**
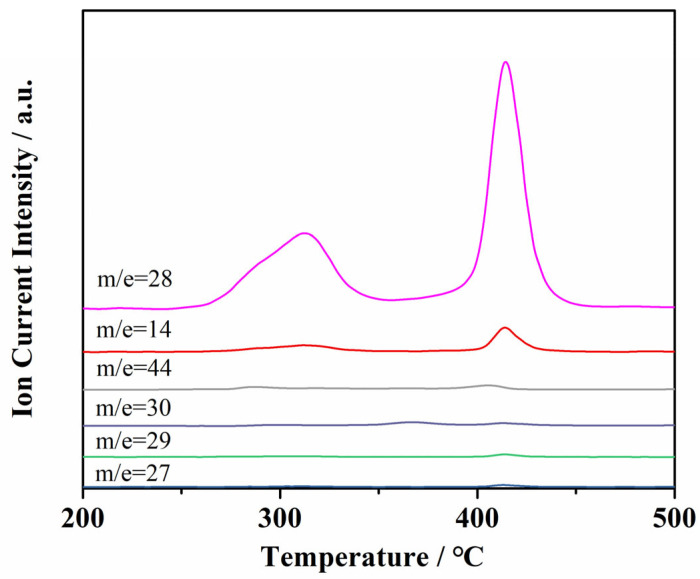
MS of gaseous products of cg-N/CNT samples obtained at a heating rate of 10 °C/min.

Figure 9 shows the MS of the volatile gas released during the thermal decomposition of cg-N/CNT at a heating rate of 10 °C/min. Charge ratios (m/z) of 28, 14, 44, 30, 29, and 27 were detected. Volatile gases with charge ratios of 28 and 14 were the main products of the thermal decomposition of cg-N/CNT at 275–375 °C and 400–440 °C, respectively. These thermal decomposition products were assumed to be gaseous and atomic nitrogen because no other signals appeared in the FT-IR spectra. That is, the major gas product of cg-N/CNT is nitrogen, which is considered a nonpolluting gas.

## 3. Materials and Methods

### 3.1. Materials

Jiangsu Xianfeng Nanomaterials Technology Co., Ltd (Jiangsu, China). supplied multiwalled carbon nanotubes (CNTs) with an inner diameter of 10–20 nm and a length of 0.5–200 µm. Sodium azide (99.5%) was obtained from Sigma-Aldrich Co., LLC (Shanghai, China). High-purity nitrogen and argon gases were provided by Xi’an Weiguang Technology Co. (Xi’an, China), which supplied multiwalled carbon nanotubes (CNTs) with an inner diameter of 10–20 nm and a length of 0.5–200 µm. 

### 3.2. Experimental Methods

Sodium azide (10 g) was dissolved in deionized water (15 g) at room temperature to prepare a saturated aqueous solution of sodium azide. The sodium azide solution (10 g) was added to a Pyrex tube containing short multiwall carbon nanotubes (200 mg). The mixture was sonicated three times for 60 min each time. The resulting suspension was coated on a quartz slide. and completely dried to yield the NaN_3_/CNT substrate.

The NaN_3_/CNT substrate (50 mg) was placed in a plasma vapor deposition chamber. A gas mixture of 50% nitrogen (N_2_) and 50% argon (Ar) was introduced into the deposition chamber at a rate of 30 mL/min for at least 30 min. The pressure in the deposition chamber was adjusted to 40 ± 2 Pa, and a power of 70 W was applied. The reaction time was at least 0.5 h. The system was gradually restored to atmospheric pressure, and plasma-synthesized samples were collected.

### 3.3. Characterization Methods

Fourier transform infrared spectroscopy (FT-IR) was performed using an infrared spectrometer (Thermo Scientific (Waltham, MA, USA), ATR50) equipped with an attenuated total reflectance (ATR) accessory. Raman spectra were obtained (HORIBA Labram (Tokyo, Japan), HR Evolution) at a 532 nm laser excitation and a spectral resolution of 2 cm^−1^. Transmission electron microscopy (TEM) images and selected area electron diffraction images were obtained with a transmission electron microscope (Talos F200i). X-ray diffraction (XRD) was conducted on an X-ray powder diffractometer (PANalytical (Almelo, The Netherlands), EMPYREAN) in the 5° to 90° range using Cu K_α_ radiation (λ = 1.54 Å) and a step size of 0.02°. XPS data were collected using an X-ray photoelectron spectrometer (Thermo Scientific (Waltham, MA, USA), K-Alpha) with Al Kα radiation (1486.6 eV) operating at 225 W.

The thermal properties of the samples were investigated by differential scanning calorimetry (METTLER TOLEDO (Giessen, Germany), DSC 2). The temperature ranged from ambient temperature to 600 °C, and argon gas at a flow rate of 50 mL/min was used. Samples weighing 1.5 ± 0.2 mg were placed in an Al_2_O_3_ crucible. Pure sodium azide and the NaN_3_/CNT samples were heated at a rate of 10 °C/min. The deposited samples were heated at rates of 5, 10, 20, and 25 °C/min to determine the kinetic parameters. DSC-TG-MS-FTIR analysis was performed on a simultaneous thermal analyzer (NETZSCH (Selb, Germany) STA449F3 STA). The deposited samples weighing approximately 1 mg were heated from 50 °C to 600 °C at a heating rate of 10 °C/min. The volatile products were analyzed by mass spectrometry (NETZSCH (Selb, Germany), QMS403) and in situ IR (Thermo Scientific (Waltham, MA, USA), NEXUS870).

## 4. Conclusions

Polynitrogen samples were synthesized under atmospheric pressure utilizing coated substrates as precursor by plasma-enhanced chemical vapor deposition on carbon nanotubes. The characterization results of the deposition product were all consistent with those of cg-N. The polnitrogen content increased nearly three times compared to the samples obtained from the powder substrate. Thermal analysis at a heating rate of 10 °C/min showed a decomposition temperature of 429 °C for the cg-N sample. The apparent activation energy of cg-N calculated by the Kissinger and Ozawa equations was 84.7 kJ/mol and 91.9 kJ/mol, respectively, with a pre-exponential constant of 12.8 min^−1^. Therefore, cg-N is an all-nitrogen material with a high energy density and ideal thermal stability, for which applications of high energy density materials are worth exploring.

## Figures and Tables

**Table 1:** The characteristic parameters for thermal decomposition processes of cg-N.

Sample Component	Heating Rate β (°C/min)	Exothermic Peak T_p_ (°C)	Apparent Activation Energy *E_a_* (kJ/mol)		Pre-Exponential Constant lnAk
			Kissinger	Ozawa	
cg-N	5	408.1	84.7	91.9	12.8
	10	429.0			
	20	464.8			
	25	476.9			
NaN_3_	5	397.6	184.9	191.2	31.8
	10	412.3			
	20	422.9			
	25	426.8			

## Data Availability

Data are contained within the article.
